# The Causes and Long-Term Consequences of Viral Encephalitis

**DOI:** 10.3389/fncel.2021.755875

**Published:** 2021-11-30

**Authors:** Karen Bohmwald, Catalina A. Andrade, Nicolás M. S. Gálvez, Valentina P. Mora, José T. Muñoz, Alexis M. Kalergis

**Affiliations:** ^1^Millennium Institute on Immunology and Immunotherapy, Departamento de Genética Molecular y Microbiología, Facultad de Ciencias Biológicas, Pontificia Universidad Católica de Chile, Santiago, Chile; ^2^Departamento de Endocrinología, Facultad de Medicina, Pontificia Universidad Católica de Chile, Santiago, Chile

**Keywords:** encephalitis, inflammation, central nervous system, viral infection, cognitive impairment

## Abstract

Reports regarding brain inflammation, known as encephalitis, have shown an increasing frequency during the past years. Encephalitis is a relevant concern to public health due to its high morbidity and mortality. Infectious or autoimmune diseases are the most common cause of encephalitis. The clinical symptoms of this pathology can vary depending on the brain zone affected, with mild ones such as fever, headache, confusion, and stiff neck, or severe ones, such as seizures, weakness, hallucinations, and coma, among others. Encephalitis can affect individuals of all ages, but it is frequently observed in pediatric and elderly populations, and the most common causes are viral infections. Several viral agents have been described to induce encephalitis, such as arboviruses, rhabdoviruses, enteroviruses, herpesviruses, retroviruses, orthomyxoviruses, orthopneumovirus, and coronaviruses, among others. Once a neurotropic virus reaches the brain parenchyma, the resident cells such as neurons, astrocytes, and microglia, can be infected, promoting the secretion of pro-inflammatory molecules and the subsequent immune cell infiltration that leads to brain damage. After resolving the viral infection, the local immune response can remain active, contributing to long-term neuropsychiatric disorders, neurocognitive impairment, and degenerative diseases. In this article, we will discuss how viruses can reach the brain, the impact of viral encephalitis on brain function, and we will focus especially on the neurocognitive sequelae reported even after viral clearance.

## Introduction

One of the most relevant neuropathology causing high morbidity and mortality worldwide is encephalitis, which is the inflammation of the brain parenchyma leading to neurological alterations (Granerod and Crowcroft, 2007; Venkatesan, 2015). Mild clinical symptoms, such as fever, headache, nausea, vomiting, confusion, and altered mental status (including personality changes) or more severe symptoms, such as seizures, weakness, hallucinations, and coma, among others are associated with encephalitis (Ferrari et al., 2009; De Blauw et al., 2020). Currently, encephalitis incidence ranges between 7 and 15 cases per 100,000 inhabitants depending on the country (Vora et al., 2014; Dubey et al., 2018; Ferreira et al., 2019). Encephalitis can affect individuals of all ages, but it is frequently observed in pediatric and elderly populations, usually caused by pathogenic infections (mainly viral infections) (Stone and Hawkins, 2007; Ferrari et al., 2009) or autoimmune responses (Dubey et al., 2018; Perlejewski et al., 2020). Viral encephalitis can be classified as either primary or secondary. Primary encephalitis requires direct infection of the brain by the pathogen, affecting one or more areas of this tissue. Secondary encephalitis occurs when the pathogen spreads from the original site of infection (i.e., lungs, kidney) to the central nervous system (CNS) (Jayaraman et al., 2018). The diagnosis of primary viral encephalitis is confirmed mainly by sampling cerebrospinal fluid (CSF), where lymphocytic pleocytosis, normal glucose levels, and high proteins levels can be found (Ekmekci et al., 2013; Rozenberg, 2013). Nowadays, the diagnose of encephalitis is performed by several methods, such as electroencephalographic (EEG) and brain magnetic resonance imaging (MRI), and by detecting several pathological changes such as hemiparesis, pyramidal signs, and seizures (Chaudhuri and Kennedy, 2002; Kennedy, 2004; Rozenberg, 2013; Ellul and Solomon, 2018; Jayaraman et al., 2018). However, to determine the possible presence of pathogenic agents causing neuropathology, additional methods are needed, such as polymerase chain reaction (PCR) assays, reverse transcription PCR (RT-PCR) assays, routine serology assays, bacterial cultures, among others (DeBiasi and Tyler, 2004; Ekmekci et al., 2013). A proper and opportune diagnosis of encephalitis leads to a better prognosis and management of the sequelae provoked by this neuropathology.

Patients that have suffered from viral encephalitis can exhibit persistent symptoms that include behavioral problems, tic disorders, recurrent headache, sleeping disorders, and motor disabilities (Michaeli et al., 2014). Several symptoms of neurocognitive impairment have also been reported as sequelae of viral encephalitis, such as attention-deficit/hyperactivity disorder (ADHD), speech disorder, and memory and learning disorders (Huang et al., 2006; Michaeli et al., 2014; Pöyhönen et al., 2021). In this article, we will first describe some relevant zoonotic viruses and their characteristics. Then, we will discuss the mechanisms reported to date used by these viruses to reach the brain and how they impact the integrity of the blood-brain barrier (BBB). We will also describe the immune response elicited in the CNS upon infection with these viruses. Following this, a characterization of the systemic immune response induced upon viral infections will be addressed. It is key to note that most viruses first induce a systemic infection and then reach the CNS. The order of the information presented in this article focuses on the impact of these viruses on the CNS rather than the systemic response. Finally, we will emphasize the long-term-sequelae described upon infection with these viruses.

## Emerging Zoonotic Encephalitis Viruses

Several human diseases, including those leading to encephalitis, might be caused by viruses that are originated or transmitted from animals to humans, called zoonotic viruses (Olival et al., 2017). These zoonotic viruses can be transmitted to humans by direct contact with fluids carrying the viral particles, such as urine, saliva, blood, or feces (Chomel, 2009). Some viruses can be transmitted through an intermediate organism (Chomel, 2009). Risk factors for zoonotic transmission include the consumption of animals and domestication of animals, among others (Chomel, 2009). Here we will describe some of the encephalitic zoonotic viruses of significant relevance worldwide.

Arboviruses are arthropod-borne viruses usually transmitted to humans by blood-feeding arthropods, such as mosquitoes, sand flies, and ticks (Beckham and Tyler, 2015; Clé et al., 2020). The group of clinically relevant neurotropic arboviruses includes, among others, West Nile virus (WNV), Japanese encephalitis virus (JEV), dengue virus (DENV), Zika virus (ZIKV), chikungunya virus (CHIKV), and tick-borne encephalitis virus (TBEV) (Beckham and Tyler, 2015; Clé et al., 2020). Arboviruses have emerged and increased due to the expansion of cities, and crowded conditions allow mosquitoes to spread this virus to a high number of humans (Baker et al., 2021). Accordingly, WNV is a mosquito-borne neurotropic virus that can cause meningitis and even lethal encephalitis in 1–2% of the infected host (Hayes and Gubler, 2006). JEV is transmitted by mosquitos, while waterbirds act as a reservoir. Also, it has been described that pigs serve an amplifying host that can be transmitted directly between this species (Ricklin et al., 2016; Zhou et al., 2021). The mortality of the JEV cases is about 30%, while 50% of the survivors developed neuropsychiatric sequels (Hsieh and John, 2020). DENV, ZIKA, and CHIKV are transmitted by the same mosquito causing encephalitis and encephalopathy, among other neurological manifestations, with an incidence between 0.5 and 20% (Hills et al., 2017; Li et al., 2017; Constant et al., 2021).

Rhabdoviruses consist of more than 175 viruses and include the rabies virus (RABV) as the only human pathogen described up to date of this group (Burrell et al., 2017). Rabies encephalitis is a widely studied zoonotic and mortal disease. The transmission of RABV is usually through the bite of an infected animal, but in some cases, it can be transmitted by direct contact with body fluids or by tissue or organ transplants (Hemachudha et al., 2013; Potratz et al., 2020b; Siepker et al., 2020). The natural reservoirs of RABV are some wild carnivores, raccoons, and bat species. Remarkably, the dog is a domestic animal that serves as the main reservoir of this virus (Burrell et al., 2017; Siepker et al., 2020; Worsley-Tonks et al., 2020).

Nowadays, among the zoonotic respiratory viruses that cause secondary encephalitis are orthomyxoviruses and coronavirus (Meseko et al., 2018; Magouras et al., 2020). Influenza virus belongs to the orthomyxovirus genus, and Influenza A virus has been described as zoonotic (Meseko et al., 2018). The subtypes of avian origin include H5, H7, and H9, while H1 and H3 have a swine-origin (Meseko et al., 2018). Significantly, the transmission of Influenza virus from humans to animals, a process known as reverse zoonosis, has also been described (Mi and Al, 2015). This is relevant for the emerging of pandemics Influenza viruses that can jump from animals to humans and backward. The pandemic of the 2009 H1N1 Influenza A virus increased influenza-associated encephalitis and encephalopathy (IAE) (Meijer et al., 2016). After this pandemic, the incidence of IAE went from 0.21 per million population to 12 per million of the symptomatic population (Meijer et al., 2016).

Human coronaviruses (HCoV) have also been implicated in the development of secondary encephalitis, despite that the primary tissue these viruses infect are the respiratory and enteric systems (Bohmwald et al., 2018; Mishra and Banerjea, 2020; Singer et al., 2021). Among the different HCoVs are HCoV-O43, HCoV-229E, Middle East respiratory syndrome coronavirus (MERS-CoV), severe acute respiratory coronavirus 1 (SARS-CoV-1), and SARS-CoV-2 (Morfopoulou et al., 2016; Bohmwald et al., 2018; Mishra and Banerjea, 2020; Kasereka and Hawkes, 2021). These viruses have a zoonotic origin in bats, and the transmission to humans originates from dromedary camels for MERS-CoV and palm civet for SARS-CoV-1 (Memish et al., 2013; Azhar et al., 2014). The transmission of SARS-CoV-2 to humans remains to be elucidated (Haider et al., 2020).

Currently, the probability of a pandemic occurring due to an emerging zoonotic virus is increasing, being a concern for public health, especially regarding encephalitis development. This pathology has demonstrated to increase the cases after the pandemic of Influenza A virus (Meijer et al., 2016).

## Viruses Inducing Encephalitis and Their Impact on the Blood-Brain Barrier

As previously mentioned, viral encephalitis is the most common cause of this neuropathology (Rozenberg, 2013; Salimi et al., 2016; Bohmwald et al., 2018; Stahl and Mailles, 2019; Chen et al., 2020). Several viruses have been described as agents leading to encephalitis, including arbovirus, rhabdoviruses, enterovirus, herpesvirus, retroviruses, orthomyxoviruses, orthopneumovirus, and coronavirus (including SARS-CoV-2), among others (Rozenberg, 2013; Salimi et al., 2016; Bohmwald et al., 2018; Stahl and Mailles, 2019; Chen et al., 2020). A viral infection must induce inflammation and damage to the brain to cause encephalitis, which can be achieved by recognizing viral particles or antigens in the CNS (Spindler and Hsu, 2012; Al-Obaidi et al., 2018; Chen and Li, 2021). One of the most common ways for viruses to reach the brain (and therefore induce this damage) is by disrupting the BBB (Spindler and Hsu, 2012; Hou et al., 2016; Bohmwald et al., 2021b; Thomsen et al., 2021).

The BBB is a physical roadblock in the interface between the CNS, the circulatory system, the immune system, and the rest of the organism (Abbott et al., 2010). This barrier is found in all vertebrates, has a highly restricted permeability, and is responsible for maintaining the microenvironment of the brain (Abbott et al., 2010). Under normal physiological conditions, the BBB protects the brain and the neurons from elements circulating in the blood that may cause damage to them, such as antibodies, toxins, immune cells, or microorganisms (Chen and Li, 2021). This barrier is mainly composed of a monolayer of brain microvascular endothelial cells found along the vascular tree [mostly kept together by tight junctions (TJ)], pericytes, and astrocytes (giving structural support to this structure) (Nikolakopoulou et al., 2019; Chen and Li, 2021). TJs in the BBB usually extend as transmembrane networks and are responsible, for the most part, for the selective permeability of this barrier (Spindler and Hsu, 2012; Hou et al., 2016; Thomsen et al., 2021). TJs are usually composed of transmembrane proteins, such as junctional adhesion molecules (JAMs), endothelial cell-selective adhesion molecule (ESAM), occludins, and claudins, with these last two anchored to the endothelium through adaptor proteins such as the zonula occludens (ZO) protein family (Daneman et al., 2010). Along these lines, the most common markers used to evaluate BBB integrity and disruption are claudins-5 and ZO-1 (Nitta et al., 2003). Remarkably, it has been described that some viruses, such as adenovirus, reovirus, and hepatitis C virus, can use TJs as an entryway to their target cells, promoting their infection. However, this entry does not necessarily disrupt the architecture of the TJ network (Barton et al., 2001; Walters et al., 2002; Evans et al., 2007).

**FIGURE 1:**
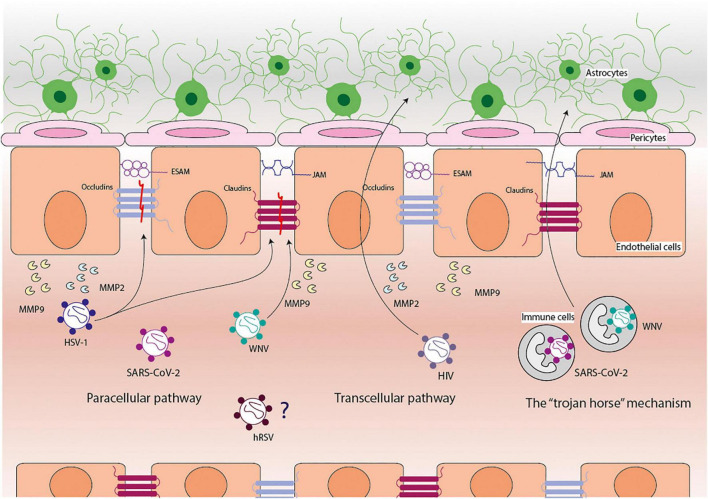
Pathways used by viruses to cross the blood-brain barrier. The blood-brain barrier (BBB) is composed of endothelial cells, pericytes, and astrocytes cells. The viruses use three principal pathways to cross the BBB: paracellular, transcellular, and the “trojan horse” mechanism. West Nile virus (WNV) can reach the central nervous system via the paracellular pathway and the “trojan horse” mechanism, disrupting claudins and promoting the secretion of matrix metalloproteinases (MMPs) 9. Herpes simplex virus (HSV-1) can reach the central nervous system via the paracellular pathway, disrupting claudins and occludins and promoting the secretion of MMP2 and MMP9. Human immunodeficiency virus (HIV) can reach the central nervous system via the paracellular and transcellular pathways and promote the secretion of MMP2 and MMP9. SARS-CoV-2 can reach the central nervous system via the paracellular pathway and the “trojan horse” mechanism; however, it has not been elucidated how and what it promotes. Human respiratory syncytial virus (hRSV) can reach the central nervous system through the disruption of the BBB, but how and what it causes this has not been elucidated yet.

Overall, there are three ways by which viruses (and most pathogens) can cross the BBB: the paracellular pathway, the transcellular pathway, and the “trojan horse” mechanism (Figure 1; Meltzer et al., 1990; Al-Obaidi et al., 2018; Chen and Li, 2021). The paracellular pathway requires viruses to move between cells of the BBB. The transcellular pathways require viruses to pass through cells of the BBB, sometimes infecting them; and the “trojan horse” mechanism is associated with the infection of circulating cells (usually immune cells) that are capable of moving across the BBB and, therefore, taking with them viruses into the brain (Meltzer et al., 1990; Al-Obaidi et al., 2018; Chen and Li, 2021). Although viruses or their molecular components do not directly disrupt the BBB, the presence of these elements induces cellular responses that cause damage to this barrier (Al-Obaidi et al., 2018).

Among the many modulators of the integrity of the BBB, the Matrix Metalloproteinases (MMPs) are endopeptidases with zinc-dependent proteolytic activities on the components of the extracellular matrix (Rosenberg, 1995). Other molecules, such as cytokines, chemokines, growth factors, lipids mediators, and free radicals, are also players in the modulation of the integrity of the BBB (Stamatovic et al., 2008). Different MMPs have been described, but the most common ones associated with cleavage of TJ are MMP2, MMP3, and MMP9 (Bojarski et al., 2004; Giebel et al., 2005; Gurney et al., 2006; Candelario-Jalil et al., 2009; Spindler and Hsu, 2012). Accordingly, these are the MMPs that are mainly secreted during neuroinflammatory events, and they can also modulate the activity of cytokines by cleaving them too (Candelario-Jalil et al., 2009). MMPs are first expressed as zymogens (the inactive state of an enzyme) and require different stimuli to be activated, such as cleavage by other MMPs or by exposure to reactive oxygen species (ROS) (Lehner et al., 2011; Spindler and Hsu, 2012). Since the presence of viral particles or antigens and the immune response elicited by the organism upon recognition of these components can induce ROS secretion, MMPs will be activated (Lehner et al., 2011; Paiva and Bozza, 2014). These pro-inflammatory processes are part of the main mechanisms associated with damage to the BBB. In this section, we will further characterize the mechanisms used by some of these viruses to reach the brain and the CNS and as to how these pathogens cause damage to the BBB.

As it was mentioned above, arboviruses are arthropod-borne viruses (Barrett and Weaver, 2012; Beckham and Tyler, 2015; Clé et al., 2020). Infections by arboviruses can result in viremia, and it has been reported that in most cases, patients are asymptomatic or exhibit symptomatology similar to flu, such as headache, high fever, muscle aches, lack of muscle coordination, disorientation, convulsions, and coma (Table 1; Barrett and Weaver, 2012; Perng and Chen, 2013; Clé et al., 2020). Moreover, it has been reported that this virus may cross the BBB by the “trojan horse” mechanism and also through the paracellular pathway without disrupting the BBB (Verma et al., 2009). Astrocytes infected with WNV *in vitro* exhibited increased RNA and protein expression of MMPs. Particularly, MMP9 has been shown to be essential for the entry of WNV into the brain, as MMP9 KO mice exhibited reduced viral loads (Wang et al., 2008; Verma et al., 2010). Remarkably, in cells culture of human brain microvascular endothelium, an increased expression of claudin-1 (and no changes on ZO-1 expression) have been reported upon infection with WNV, consistent with the lack of BBB disruption described *in vitro* (Verma et al., 2009). Different reports have shown that the capsid from WNV can induce the disruption and loss of claudin-2 and -3 in mice *in vivo* and increase the permeability of the BBB, while ZO-1 and occludin remain unchanged (Medigeshi et al., 2009). Since the differences of these studies are notorious (i.e., the first one is *in vitro* with human cells, while the second one is *in vivo* with mouse models), further studies are still required to understand the capacity of WNV to modulate the permeability and disrupt the integrity of the BBB.

**TABLE 1:** Viral agents causing encephalitis and symptomatology.

Classification	Virus	Symptomatology
Arboviruses	WNV	Headache, high fever, muscle aches, lack of muscle coordination, disorientation, convulsions, and coma.
	JEV	
	DENV	
	ZIKV	
	CHIKV	
	TBEV	
Rhabdoviruses	RABV	Fever, hypersalivation, hydrophobia, swallowing difficulty, throat pain, aerophobia, neurological signs, coma, and multiple organ failure
Enteroviruses	EV71	Fever, headache, respiratory illness, vomiting, diarrhea, autonomic nervous system dysregulation, and cardiopulmonary failure.
	RV	
Herpesviruses	HSV-1	Fever, headache, seizure, focal neurological deficits, and general encephalopathy.
	HSV-2	
Retroviruses	HIV	Mental concentration, leg weakness, slowness of hand movement, personality changes, apathy, and social withdrawal.
Orthomyxoviruses	Influenza virus	Febrile seizures, convulsions, ataxia, and status epilepticus
Orthopneumovirus	hRSV	Headache, dizziness, confusion, hypogeusia, and hyposmia
Coronaviruses	HCoV-O43	
	HCoV-229E	
	SARS-CoV-1	
	SARS-CoV-2	

Herpesviruses have been described as a common cause of sporadic viral encephalitis worldwide, with herpes simplex virus-1 (HSV-1) and herpes simplex virus-2 (HSV-2) most frequently found in CSF samples from patients with viral encephalitis (mainly neonates and adults) (Marcocci et al., 2020; Aksamit, 2021). The primary infection of HSVs occurs through direct contact of the host mucosal membranes or damaged skin with infected fluids. This virus can then reach and infect sensory neurons, leading to secondary encephalitis (Bradshaw and Venkatesan, 2016; Marcocci et al., 2020; Acuña-Hinrichsen et al., 2021). The clinical signs described in patients with HSV encephalitis (HSE) are fever, headache, seizure, focal neurological deficits, and general encephalopathy (Table 1; Bradshaw and Venkatesan, 2016). HSV-1 is one of the most prevalent viruses worldwide, with over 70% of the population carrying this virus (Whitley, 2006). This neurotropic virus is usually found in a latent state in the trigeminal ganglia and the olfactory bulb after primary infection of the target mucosa (Feldman et al., 2002). Unlike other viruses addressed here, it is thought that HSV-1 gain access to the CNS through retrograde transport into the nerve fibers on the sites indicated above, therefore not disrupting the BBB on a first instance (Figure 1; Jennische et al., 2015). However, the presence of this virus may cause HSE, which in turn will promote damage to the BBB, manifesting clinical signs, such as headaches, fever, seizures, as well as cognitive and behavioral sequelae (Steiner, 2011; Jennische et al., 2015; Liu et al., 2019). The specific mechanisms underlying the disruption of the BBB by HSV-1 during HSE have not been elucidated yet, but some reports have made advancements in this field. For instance, increased activity of MMP2 and MMP9 has been reported in HSE mouse models using either HSV-1 or mouse adenovirus (MAV)-1, which led to disruption of TJs and components of the extracellular matrix (Zhou et al., 2010; Ashley et al., 2017). Remarkably, infection with MAV-1 resulted in increased permeability of the BBB, which could not be related to inflammation (Gralinski et al., 2009). *In vitro* infection of primary cultures of the mouse brain, endothelial cells with HSV-1 resulted in a downregulation of occludin and claudin-5, along with significant cell apoptosis and Golgi apparatus fragmentation (Deng et al., 2018; He et al., 2020). Remarkably, infection of HeLa cells with HSV-2 resulted in no differences in the expression of ZO-1, although this has not been evaluated for HSV-1 (Miezeiewski et al., 2012). Most of these results support the notion that HSV-1 can disrupt the BBB. However, more detailed analyses are required to address further the specific mechanisms shown by this virus to disrupt this barrier, such as the impact on TJ components like ZO-1.

Human immunodeficiency virus (HIV) is a highly prevalent virus that belongs to the retroviruses group and is the agent causative of the acquired immunodeficiency syndrome (AIDS) and can also cause encephalitis (Simon et al., 2006; Maartens et al., 2014). Transmission of HIV is through the direct contact of infected body fluids (such as blood, semen, breast milk, and vaginal secretions) [Deeks et al., 2015; German Advisory Committee Blood (German Advisory Committee Blood (Arbeitskreis Blut), Subgroup ‘Assessment of Pathogens Transmissible by Blood’, 2016)]. One of the most critical aspects of the neurological impact of HIV infection is the HIV-associated neurocognitive disorder (HAND). HAND is a pathology characterized by a deficit of mental concentration, leg weakness, slowness of hand movement, mood alterations, apathy, and social withdrawal (Table 1; Ghafouri et al., 2006; Wang et al., 2020). HIV is also one of the most studied viruses that significantly impact the BBB (Strazza et al., 2011; Spindler and Hsu, 2012; Caligaris et al., 2021). This virus can move through the BBB by transcellular and paracellular diapedesis of infected lymphocytes, leading to barrier damage and encephalitis (Ivey et al., 2009). *In vitro* studies showed that PBMCs infected with HIV-1 exhibited enhanced capacities to cross brain-derived micro vascularity in primary cultures, compared to uninfected PBMCs (Eugenin et al., 2006). The presence of HIV-1-infected PBMCs also led to increased expression of MMP-2 and MMP-9, along with altered expression of TJ (Eugenin et al., 2006).

Human immunodeficiency virus expresses different proteins that are part of the viral particle, and some of those have been shown to play a role in the modulation of the BBB integrity. The protein gp120 is an envelope protein from HIV-1 with various effects in the host [German Advisory Committee Blood (German Advisory Committee Blood (Arbeitskreis Blut), Subgroup ‘Assessment of Pathogens Transmissible by Blood’, 2016)]. *In vitro* and *in vivo* studies showed that the presence of gp120 increased the permeability to albumin of the BBB (a protein that in normal conditions does not cross the BBB) (Cioni and Annunziata, 2002; Strazza et al., 2011). This viral antigen also was responsible for reducing the expression of ZO-1 and occludin in human brain endothelial cultures *in vitro* (Kanmogne et al., 2005). The addition of Tat (an HIV protein associated with transactivation, enhancement of initiation and elongation of viral transcription [German Advisory Committee Blood (Arbeitskreis Blut), Subgroup ‘Assessment of Pathogens Transmissible by Blood’, 2016] to human brain microvascular cultures resulted in altered TJ expression, inflammation, and the expression of proteolytic enzymes (Strazza et al., 2011). This protein also reduced the expression of occludin and enhanced its cleavage by MMP9 (increasing the mRNA levels, protein levels, and enzymatic activity of this MMP) in these cultures (Xu et al., 2012; Marino et al., 2020). Nef is an HIV-1 protein with roles described in modulating the expression of MHC-I and CD4 in lymphocytes [Haller et al., 2014; German Advisory Committee Blood (Arbeitskreis Blut), Subgroup ‘Assessment of Pathogens Transmissible by Blood’, 2016]. This protein increases the sensibility of astrocytes to ROS *in vitro* cultures. Therefore, Nef can modulate the expression and activity of enzymes, such as MMP9 (Lévêque et al., 2004). Different reports have also evaluated the role of HIV *in vivo*. For instance, reduced expression of ZO-1, occludin, and claudins have been described in post-mortem samples from HIV-infected humans exhibiting encephalitis or dementia (Dallasta et al., 1999; Boven et al., 2000). Accordingly, pericytes in the BBB can also be infected by HIV in mice, and the expression of occludin by these cells modulates the transcription of HIV (Castro et al., 2016; Bertrand et al., 2019). Administration of gp120 to rats resulted in increased levels of ROS, which led to reduced expression of claudin-5 and increased expression of MMP2 and MMP9 (Louboutin et al., 2010). Mice treated with Tat also showed a decreased expression levels of ZO-1 and accumulation of inflammatory immune cells (Marino et al., 2020). Finally, administration of Nef to rats also led to an MMP9-mediated BBB disruption, which could be reverted by previous treatment with inhibitors for this enzyme (Sporer et al., 2000). Altogether, several studies are addressing the capacities of HIV to modulate and disrupt the components of the BBB, and further studies should focus on elucidating the molecular mechanisms and finding therapeutic approaches to prevent BBB disruption.

Respiratory viruses also have the ability to produce secondary encephalitis (Bohmwald et al., 2018; Mastrolia et al., 2019; Yuan et al., 2019). For instance, orthomyxoviruses such as Influenza virus have been detected in CSF from patients with respiratory symptoms and mild altered mental state, vertigo, or febrile seizures (Table 1; Bohmwald et al., 2018; Mastrolia et al., 2019; Yuan et al., 2019). Human respiratory syncytial virus (hRSV), an orthopneumoviruses, has also been associated with secondary encephalitis (Millichap and Wainwright, 2009; Bohmwald et al., 2018; Kawasaki et al., 2019). Among the clinical signs related to neurological complications due to hRSV infection are febrile seizures, convulsions, ataxia, and status epilepticus (Table 1; Bohmwald et al., 2018).

Virtually every human in the world has been infected with hRSV (Calvo et al., 2008; Gálvez et al., 2017; Bohmwald et al., 2018; Ali et al., 2020). This virus is one of the main responsible for causing acute respiratory tract infections in developing and developed countries, with a significant burden on newborns, infants, and the elderly (Gálvez et al., 2017; Bohmwald et al., 2018; Mandi et al., 2021; Tabor et al., 2021). Reports have shown that hRSV can disrupt the BBB, as seen in Evans Blue (EB) permeability assays (Saunders et al., 2015; Bohmwald et al., 2021b). EB is a molecule that binds to albumin (a protein found circulating in the blood) in normal physiological conditions and cannot cross the BBB (Espinoza et al., 2013; Saunders et al., 2015; Bohmwald et al., 2021b). The presence of both EB in the brain (meaning that albumin crossed the BBB), viral antigens, and genetic material indicate that this virus can disrupt the integrity of the BBB (Espinoza et al., 2013; Bohmwald et al., 2021b). Remarkably, BBB disruption and the presence of hRSV in the CNS have been shown to impact different behavioral and cognitive capacities in mouse models (Espinoza et al., 2013; Bohmwald et al., 2021b). Further studies are required to evaluate the mechanisms underlying BBB disruption by hRSV, i.e., changes on TJ components, such as ZO-1 or in the expression of MMPs.

Regarding the HCoVs, HCoV-O43, HCoV-229E, SARS-CoV-1, and SARS-CoV-2 have been detected in patients exhibiting neurological symptoms, such as headache, dizziness, confusion, hypogeusia, and hyposmia (Table 1; Morfopoulou et al., 2016; Bohmwald et al., 2018; Mishra and Banerjea, 2020; Kasereka and Hawkes, 2021). Coronavirus disease 2019 (COVID-19) is the disease caused by SARS-CoV-2, the virus responsible for the pandemic affecting the whole world since December 2019 (Zhu et al., 2020). This pandemic has caused the death of over four million people, although vaccines and treatments are just starting to control the spread and the severity of this virus (Dong et al., 2020; WHO, 2020). As previous coronaviruses were known to have an impact on the CNS, and several neurological sequelae have been reported after SARS-CoV-2 infection in humans, a significant number of studies have focused on the impact of SARS-CoV-2 in the brain and the BBB (Bohmwald et al., 2018; Desforges et al., 2019; Fiani et al., 2020). Studies using HCoV-OC-43 and HCoV-229E have shown that these coronaviruses can enter the brain through the paracellular pathway and the “trojan horse” mechanism; therefore, SARS-CoV-2 could be using the exact mechanisms, as genetic material from this virus has also been found in post-mortem brain samples (Desforges et al., 2007; Li et al., 2020; Paniz-Mondolfi et al., 2020; Zubair et al., 2020). *In vitro* use of human pluripotent stem cell-derived brain organoids have shed some light on the infective capacity of SARS-CoV-2 at the CNS (Pellegrini et al., 2020). Among the many cell types found in these organoids, only mature choroid plexus cells were infected, while neurons and other cell types were not (Pellegrini et al., 2020). Remarkably, a decrease in the expression of claudin-5 was observed on the epithelium of SARS-CoV-2-infected cells, which could be associated with the BBB disruption (Pellegrini et al., 2020). Although further studies are required to characterize the impact of this novel virus on the BBB function and the modulation of other elements, such as occludins and MMPs, it has been recently shown that the envelope protein (E) of SARS-CoV-2 can interact with ZO-1 on TJ (Shepley-McTaggart et al., 2021). This phenomenon likely plays a role in disrupting the BBB function (Shepley-McTaggart et al., 2021).

As it was previously mentioned, RABV is the most studied zoonotic neurotropic virus with symptoms such as fever, hypersalivation, hydrophobia, swallowing difficulty, throat pain, aerophobia, neurological signs, coma, and multiple organ failure (Table 1; Hemachudha et al., 2013; Sharma et al., 2015). Besides, RABV neuroinvasion is through the infection of peripheral neurons located in the bite site, altering the BBB permeability to increase its replication in the CNS (Qingqing et al., 2014). Indeed, only a laboratory-attenuated RABV infection decreases the expression of claudin-5, occludin, and ZO-1, which increase the BBB permeability, allowing the infiltration of the immune cells into the brain (Qingqing et al., 2014). These effects were not observed with the WT RABV infection, suggesting that this virus evaded the immune response maintaining the BBB permeability intact (Qingqing et al., 2014).

Enteroviruses infections are mainly transmitted by the fecal-oral route, and viral replication usually occurs in the gastrointestinal tract (Chen et al., 2020). Several enteroviruses capable of causing encephalitis, such as poliovirus (PV) and enterovirus 71 (EV71) (Huang et al., 2006; Huang and Shih, 2015; Chen et al., 2020). Generally, enterovirus infections are asymptomatic, but they can also cause a wide range of clinical manifestations, such as fever, headache, respiratory illness, vomiting, diarrhea, autonomic nervous system dysregulation, and cardiopulmonary failure (Table 1; Huang and Shih, 2015; Chen et al., 2020).

Autoimmune encephalitis has also been reported in several cases (Lancaster, 2016). This neuropathology is challenging to diagnose due to similar clinical analyses performed with other autoimmune diseases or viral encephalitis (Lancaster, 2016). Autoimmune encephalitis can be classified into two groups. In the first one, known as paraneoplastic syndromes, autoantibodies attack intracellular proteins such as anti-Hu, anti-Yo, and anti-Ri (Lancaster, 2016; Hermetter et al., 2018), while in the second group, autoantibodies are directed against extracellular epitopes of ion channels, neuronal receptors, or synaptic proteins including anti-*N*-methyl D-aspartate-receptor (NMDAR) and metabotropic glutamate receptor 5 (mGluR5) (Lancaster, 2016; Hermetter et al., 2018). Importantly, encephalitis caused by damage to NMDAR exhibits symptoms, such as behavioral changes, hallucinations, seizures, amnesia, and movement disorders (Prüss et al., 2010). Antibodies against NMDAR have been found in serum from patients who were diagnosed or recovered from HSE, CHIKV, TBEV, Influenza virus, WNV, enteroviruses, and SARS-CoV2, among others (Adang et al., 2014; Cavaliere et al., 2019; Karagianni et al., 2019; Sahar et al., 2019; Alvarez Bravo and Ramió i Torrentà, 2020; Nóbrega et al., 2020). One crucial aspect to consider is that secondary encephalitis can be caused by an infection of the CNS and a systemic inflammation where normal brain functions are affected (Hosseini et al., 2018, 2021).

## Immune Response at the Central Nervous System During Viral Infections

As mentioned above, the first step to initiate viral encephalitis is the BBB disruption. Once the virus enters the CNS, the first line of defense consists of microglia, which are the primary innate immune response of the CNS (Kofler and Wiley, 2011; Ousman and Kubes, 2012; Lenz and Nelson, 2018; Bohmwald et al., 2021a). In the brain, microglia play an essential role in maintaining homeostasis, supporting neurons by the secretion of neurotrophins and growth factors, in addition to their contribution to the immune response against pathogens (Kofler and Wiley, 2011; Ousman and Kubes, 2012; Lannes et al., 2017a; Chhatbar and Prinz, 2021). Microglia are susceptible to being infected by several viruses, including WNV, JEV, DENV, ZIKV, HIV, and HSV-1, among others (Jhan et al., 2017; Wang et al., 2018). The astrocytes are one of the most abundant cell types in the brain and plays several roles in brain homeostasis, such as regulation of the ion balance and neurotransmitters, support of the neuronal synapses, and maintenance of BBB permeability (Colombo and Farina, 2016; Soung and Klein, 2020; Bohmwald et al., 2021a). Like microglia, activated astrocytes can secrete factors that allow immune cell recruitment into the injured area, promoting the amplification of the neuroinflammation (Farina et al., 2007). Astrocytes are permissive to infection by WNV, JEV, ZIKV, TBEV, HSV-2, HIV, hRSV, and SARS-CoV-2 (Palus et al., 2014; Huang et al., 2018; Potokar et al., 2019; Crunfli et al., 2020; Lutgen et al., 2020; Bohmwald et al., 2021b; Słońska et al., 2021). Neurons can have different functions, depending on their location in the brain. For instance, neurons close to the BBB contribute to the regulation of blood flow and secrete factors that promote angiogenesis, among others (Bohmwald et al., 2018; Rhea and Banks, 2019). The pyramidal glutamatergic neurons function at the hippocampus is mainly associated with learning, memory, and emotions (Wheeler et al., 2015; Spencer and Bland, 2019). For several viruses, including WNV, JEV, ZIKV, TBEV, HSV, HIV, Influenza virus, hRSV, and SARS-CoV-2, neurons are the primary infection target in the brain (Kopp et al., 2009; Wang et al., 2016; Bohmwald et al., 2018; Klein et al., 2019; Clé et al., 2020). The infection of neurons promotes cell damage, death, the secretion of cytokines that induce immune cell recruitment, and alterations in neurocognitive processes (Bílı et al., 2015; Bohmwald et al., 2018; Klein et al., 2019).

**FIGURE 2:**
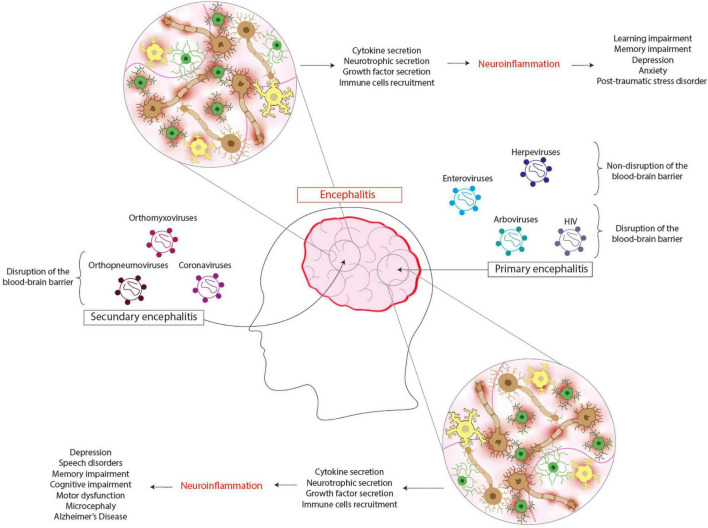
Viral encephalitis leads to several neurologic sequelae. Primary encephalitis is caused by viruses that infect the brain as their principal target, which can be found in arboviruses, enteroviruses, herpesviruses, and HIV. The infection of cells from the central nervous system induces the cytokine, neurotrophic, and growth factor secretion and the recruitment of immune cells. It is thought that this response can contribute to neuroinflammation, leading to neurologic sequelae such as depression, speech disorders, memory, and cognitive impairment, motor dysfunction, microcephaly, and even Alzheimer’s disease. Secondary encephalitis is caused by viruses that do not infect the brain as their principal target, which can be found in orthomyxovirus, orthopneumoviruses, and coronaviruses. The infection of cells from the central nervous system induces the cytokine, neurotrophic, and growth factor secretion and the recruitment of the immune cells. This response contributes to neuroinflammation, leading to neurologic sequelae such as learning and memory impairment, depression, anxiety, and post-traumatic stress disorder.

Studies evaluating WNV encephalitis have shown that human fetal cultures of microglia are less permissive to viral infections than human fetal cultures of neurons and astrocytes (Cheeran et al., 2005). However, poor viral infection promoted microglia activation and TNF-α secretion at an early stage of the infection and, later, IL-6, CCL2, CCL5, and CXCL10, which was not observed when inactivated WNV was administered to these cells (Cheeran et al., 2005). Moreover, increased susceptibility to infection has also been shown during the infection with an attenuated strain of WNV in mice lacking IL-34 (absent of microglia but not bone marrow-derived macrophages) therefore confirming the protective role of microglia (Wang et al., 2012). Additionally, WNV-infection of astrocytes promotes the expression of pro-inflammatory mediators and the release of neurotoxins (van Marle et al., 2007). Indeed, neuron death induced by WNV is not only by direct infection, but it is also promoted by infected astrocytes (Figure 2; van Marle et al., 2007; Potokar et al., 2019).

One of the models used to study viral infection of microglia *in vitro* is through the use of human peripheral blood monocytes, which can differentiate into monocyte-derived microglia after culture with a serum-free medium containing M-CSF, GM-CSF, NGF, and CCL2 (Etemad et al., 2012; Rai et al., 2020). Studies performed in JEV-infected human blood monocytes-derived microglia model showed a contact-dependent cell-to-cell transmission, which involves the CX3CR1-CX3CL1 axis allowing an early infection of neurons (Lannes et al., 2017b,^2019^). It has been proposed that microglia can be the reservoir for JEV (Thongtan et al., 2010). In mouse microglial cells (BV2), exposure to JEV resulted in 95% of cells being infected after 24 h, and this remained constant until 5 days post-infection (Thongtan et al., 2010). JEV-infected microglia were activated, and after they were sub-cultured 2-3 times per week for 16 weeks, the microglial cells showed a long-term infection (Thongtan et al., 2010). Notably, the activation of microglia promoted an increase in the expression of CCR2, tumor necrosis factor-alpha (TNF-α), and interferon gamma (IFN-γ) (Singh et al., 2020). The inhibition of CCR2 in JEV-infected microglia reduces their activation and, consequently, the expression of pro-inflammatory cytokines (Singh et al., 2020). Similarly to WNV and TBEV, JEV can infect astrocytes and induce the secretion of pro-inflammatory molecules, such as CXCL10 and TNF-α and also type I IFN, which contribute to limiting viral spreading in these cells (Figure 2; Lindqvist et al., 2016; Potokar et al., 2019). Additionally, it has been described that JEV infects dopaminergic neurons and induces the secretion of high dopamine levels, which increases dopamine receptor 2 (DR2) involved in viral entry (Simanjuntak et al., 2017). Also, JEV infection of neurons promotes the activation of NMDAR, which increases the release of glutamate and pro-inflammatory cytokines, promoting neuronal death (Chen et al., 2018).

It is known that ZIKV impairs normal neurodevelopment due to the infection of neural cells. However, it can also infect microglia, promoting the secretion of pro-inflammatory cytokines, such as TNF-α and IL-6, and therefore inhibiting neuronal differentiation of neuronal precursor cells (NPCs) (Wang et al., 2018). Fetal astrocytes are more permissive to ZIKV infection than fetal neurons or NPCs (Huang et al., 2018). In a human astrocyte cell line (U251), viral infection induces protein expression changes associated with several signaling pathways, including axonal guidance and the expression of genes involved in neurological disorders (Sher et al., 2019; Shereen et al., 2021). Furthermore, ZIKV infection of hippocampal neurons promotes changes in the expression of molecules involved in the communication between astrocytes and neurons, such as NMDAR and neurotrophins (Bobermin et al., 2020).

Studies performed in neurons and astrocytes differentiated from human fetal neuronal progenitors (hNPCs) showed that this cell type is permissive to be infected by TBEV (Fares et al., 2020). Viral infection promotes neuron and astrocyte death and the production of type I IFNs, which is implicated in the control of viral replication and cell protection (Selinger et al., 2017; Fares et al., 2020).

Concerning the RABV neurotropism, it is well known that the infection of motor neurons and dorsal root ganglia (DRG) sensory neurons help this virus to spread from the site of the infection to the CNS through an axonal anterograde and retrograde transport (Velandia-Romero et al., 2013; Davis et al., 2015; MacGibeny et al., 2018; Potratz et al., 2020a). Most studies have shown that infection of neurons with laboratory-adapted RABV can induce more neuronal apoptosis than WT RABVs (Fernandes et al., 2011). Although neurons are the primary target of infection of this virus, several reports show that astrocytes can also be infected with different RABVs strains (Tian et al., 2018; Potratz et al., 2020a). In this line, RABV Tha (the WT strain) and Th2P-4M (a less virulent strain) can replicate more efficiently in human neuroblastoma cell lines (SK-N-SH) as compared to astrocyte-like (SVGp12) and microglia-like (HMC3) cell lines. It has also been reported that RABV only infects human neural stem cells (hNSC) differentiated into hiNeurons and hiAstrocytes, while it does not infect iPSC differentiated into hiMicros (Feige et al., 2021). Interestingly, it has been reported that glial cells display a protective role when co-cultured with infected neurons, reducing and limiting the infection of RABV. Additionally, glial cells differentially express and secrete cytokines such as IL-6, CCL5, and CXCL10, as part of the innate immune response against RABV, which varies depending on the viral strain (Tian et al., 2018; Feige et al., 2021).

Interestingly, HSV not only infects neurons but can also establish latency on these cells as well as in astrocytes (Marcocci et al., 2020; Słońska et al., 2021). HSV-1-infected cortical neurons exhibit an altered expression of dendritic spine proteins as well as their distribution and changes in response to a glutamate stimulation altering the normal function of these cells (Acuña-Hinrichsen et al., 2021). Importantly, HSV-1-infected NCSs present impairment in their growth, proliferation, and neuronal differentiation, which can cause neurodevelopment disorder observed in neonatal HSV-1 infections (Qiao et al., 2020). On the other hand, microglia also are permissive to HSV-1 infection, but the viral replication is limited due to the secretion of antiviral molecules, such as TNF-α, IL-1β, CCL5, and CXCL10, suggesting a protective role for these cells (Lokensgard et al., 2001). However, the chronic infection of HSV-1 induces a prolonged activation state of microglia characterized by the secretion of chemokines including CCL2, CCL5, and CXCL10 that allow peripheral immune cell infiltration into the brain (Figure 2; Wang et al., 2019).

Neuroinvasion of HIV occurs in the acute stage of the infection, where the virus can infect astrocytes and microglia, while the infection of neurons remains controversial (Kovalevich and Langford, 2012; Wallet et al., 2019; dos Reis et al., 2020; Lutgen et al., 2020). HIV can infect astrocytes in the brain and then egress to the peripheral organs through the infection of CD4^+^ T cells (Lutgen et al., 2020). Additionally, it has been reported that microglia may act as the main reservoir for HIV in the brain due to in these cells, there is no evidence of cytopathic effect, lysis, or cell apoptosis (Wallet et al., 2019). Similar to other viral infections, the activation of microglia promotes the secretion of pro-inflammatory cytokines, such as TNF-α, IL-6, IL-10, and CXCL8 (Figure 2; Tatro et al., 2014). Regarding the HIV latency on microglia, it has been described that dopaminergic and GABAergic but not cholinergic motor neurons can control the expression of HIV and prevent the reactivation of this virus (Alvarez-Carbonell et al., 2019). Conversely, HIV-infected microglia induce neuronal damage (Alvarez-Carbonell et al., 2019).

Recently, studies in mice described that hRSV could infect several brain cells, such as microglia, astrocytes, and neurons, among others (Bohmwald et al., 2021b). Studies in primary mouse astrocyte cultures have shown that hRSV infection promotes cellular activation, increasing production of nitric oxide (NO) as well as the expression of GFAP, which increased both at the levels of GFAP per astrocyte and the number of activated astrocytes (Eng et al., 2000; Bohmwald et al., 2021b). Besides, hRSV-infected astrocytes secrete pro-and anti-inflammatory cytokines during the acute phase of infection, which can be the source of cytokines observed in the brain of hRSV-infected mice (Figure 2; Bohmwald et al., 2021b). Further experiments are required to address the effect of hRSV infection in microglia and neurons.

## Systemic Immune Response Against the Viral Infection

Neurotropic viruses can reach the brain and promote a local infection in this tissue. However, most of these viruses have a primary infection site outside the CNS, where the systemic immune response is elicited (Luethy et al., 2016). From there, neurotropic viruses can reach the CNS through different pathways, such as the infection of peripheral nerves or its dissemination through the bloodstream (viremia) that allows the virus to cross the BBB as described earlier (Luethy et al., 2016). During these events, the infection caused by these viruses in the primary tissue target, including the CNS, can lead to a systemic immune response against them, which is characterized by the increase of pro-inflammatory cytokines and leukocytes infiltrating different tissues besides the infection site, such as the CNS (Libbey and Fujinami, 2014; Zotova et al., 2016; Filgueira et al., 2021). This section will discuss the systemic immune response induced and this effect on the clearance of the viral neuroinvasion.

It is known that patients with viral encephalopathies exhibit specific characteristics, such as abnormal concentrations of lymphocytes in CSF (Stone and Hawkins, 2007). In the case of arboviruses, the neuroinvasion of WNV induces the recruitment of peripheral T cells and monocytes, which can infiltrate the brain (Fulton et al., 2020). Additionally, local infection with arboviruses promotes the recruitment of CD8^+^ T cells from the periphery into the brain due to the continued activation of astrocytes (Soung and Klein, 2018). Even though the infiltration of T cells into the brain promotes the clearance of viruses, such as WNV and ZIKV, studies in mice have shown that these cells are responsible for learning defects and destruction of presynaptic, postsynaptic terminals, and neuronal apoptosis (Garber et al., 2019). Infection with EV71 can promote the infiltration of peripheral immune cells, such as T cells, B cells, monocytes, and dendritic cells, into the CSF (Chen et al., 2020). Interestingly, the expression of CD40L on T cells and the secretion of IL-4 decreases in EV71-infected infants (Chen et al., 2020).

The infection with the laboratory strain of RABV has been characterized by the inflammation of the CNS, where it could be seen an increase of different inflammatory molecules such as cytokines, chemokines, and IFNs (Wang et al., 2005). Since the pro-inflammatory molecular response is responsible for recruiting leukocytes into the infected tissue, it has been suggested that these molecules are responsible for the infiltration of different immune cells, such as dendritic cells, macrophages, neutrophils, and lymphocytes, into the CNS (Kuang et al., 2009; Luo et al., 2020). Even more, the presence of CD4^+^ and CD8^+^ T cells found on the brain of patients infected with RABV represented the majority of the population within the brain that were undergoing apoptosis (Fernandes et al., 2011). These last observations suggest that RABV promotes the apoptosis of T lymphocytes to avoid clearance by the immune system.

As discussed previously, arboviruses can induce peripheral T cell infiltration through the activation of astrocytes, and herpesviruses seem to have a similar mechanism to recruit immune cells into the brain (Marques et al., 2008). Studies with HSV-1 on murine models have shown that brain infection induces the activation of microglial cells, and following their activation, infiltration of immune cells can be observed (Marques et al., 2008). These data suggest that the activation of microglial cells can induce the recruitment of immune cells from the periphery (Marques et al., 2008). Among immune cells recruited into the brain, macrophages and neutrophils can be found during the first days of infection, and T cells can be found even 30 days post-infection, which correlates with the activation of microglial cells that lasted up to 30 days post-infection (Marques et al., 2008). Consistently, brain infection with HIV promotes the recruitment of immune cells from the periphery, such as macrophages, dendritic cells, and T cells, following the activation of microglial cells (Ousman and Kubes, 2012).

Reports of patients with encephalopathies due to local infection with Influenza virus in the brain have shown an increased transcription for IL-6, IL-10, and TNF-α in immune cells from the peripheral blood (Kawada et al., 2003). Both IL-6, IL-10, and sTNF-R1 levels correlate with the peripheral blood concentration of these cytokines, and death was usually reported in patients with extremely high concentrations of these cytokines (Kawada et al., 2003). Interestingly, these studies suggest that encephalopathies increase the pro-inflammatory response in these patients, compared to patients only with pulmonary symptoms due to Influenza virus infection (Kawada et al., 2003). Additionally, infants with encephalopathies due to Influenza virus infection can exhibit leukopenia in some cases, while leukocytosis could be observed in others (Mastrolia et al., 2019). Infants diagnosed with encephalopathies due to hRSV infection exhibited a systemic increase of cytokines, and slight increases in CD4^+^ and CD8^+^ T cells numbers have been detected (Ayukawa et al., 2004; Bohmwald et al., 2018). Infection with SARS-CoV-2 promotes T cell and monocyte infiltration into the CNS, where microglial cells may be responsible for the recruitment of these peripheral immune cells (Pacheco-Herrero et al., 2021). Similarly, SARS infections promoted T cells and monocytes infiltration into the CNS (Gu et al., 2005; Wu and Tang, 2020). These data suggest a general infiltration of these cell types from the peripheral blood into the CNS during encephalitis due to coronaviruses.

Non-neurotropic viruses can also induce encephalopathies through the systemic immune response. Non-neurotropic viruses such as Influenza virus (H1N1) increase the levels of B cells, CD8^+^ T cells, and monocytes but decrease the number of regulatory T cells (Frisullo et al., 2011; Maria et al., 2018). Interestingly, infection with this virus increases the activation of microglial cells, leading to the recruitment of immune cells into the brain (Sadasivan et al., 2015).

## Long-Term Sequelae Due to Viral Infections

The inflammation previously described due to neurotropic viruses can induce several neurologic diseases, such as encephalitis that can lead in some cases to neuronal death (Griffin, 2011; Klein et al., 2017). Thus, a viral infection of the brain can induce permanent neurologic damage, which is more common after viral encephalitis (van den Pol, 2009; Klein et al., 2017). The sequelae reported after viral encephalitis can involve cognitive impairments, motor dysfunction, and epilepsy, where the cases of epilepsy have been described up to 20% in the survivors of viral encephalitis (Libbey and Fujinami, 2011; Schmidt et al., 2011). In this section, the long-term sequelae because of the viral neuroinvasion will be discussed.

Patients diagnosed with viral encephalitis due to WNV tend to develop long-term neuro-pathologies, such as depression, speech disorders, memory, and cognitive impairment, motor dysfunction, and in some rare cases, epilepsy (Klein et al., 2017; Fulton et al., 2020; Zheng et al., 2020). In these patients, cognitive symptoms such as memory loss, loss of concentration, depression, irritability, and confusion can be detected from 6 to 18 months after the initial infection (Klee et al., 2004; Klein et al., 2017). Studies performed in mice have shown that infection with WNV can interfere with the neurological synapsis and promote neuronal dysfunction and astrocytosis, leading to spatial learning deficits and memory loss (Klein et al., 2017; Fulton et al., 2020). Similar to the reports for WNV, JEV infection can cause a decrease in IQ, speech disorders, memory, and adaptive behavioral impairment, even 2 years after the initial infection (Figure 2; Yin et al., 2015). Additionally, it has been reported that the infection with JEV can promote the development of epilepsy, possibly due to the secretion of pro-inflammatory molecules from activated microglial cells, their proliferation, and the generation of microglial nodules (Ghoshal et al., 2007; Zheng et al., 2020). Other arboviruses like ZIKV can lead to sequelae on the offspring, such as microcephaly and brain anomalies, after infecting pregnant women (Figure 2; Panchaud et al., 2016). Even more, the microcephaly caused by the infection with ZIKV can lead to epileptic activity in more than 50% of the cases (Zheng et al., 2020). Sequelae are generally associated with cognitive symptoms within the arboviruses, and only ZIKV causes symptoms in newborns (Panchaud et al., 2016).

Infants with encephalitis due to infection with EV71 exhibited symptoms such as epilepsy, cerebellar dysfunction, cranial nerve palsy, neurodevelopmental delay, cognitive disorders, and ADHD (Huang et al., 2006; Chang et al., 2019; Tseng et al., 2020). Interestingly, cognitive impairments are related to the severity of the local infection on the brain by EV71, along with the age of infection, as infants with severe symptoms during the infection presented higher rates of neuropathological sequelae (Figure 2; Chang et al., 2019). This observation suggests that enteroviruses can cause massive neuron damage in young patients following severe symptoms during infection.

Studies of patients who had been diagnosed with viral encephalitis due to HSE have demonstrated sequelae such as speech disorders, memory, and cognitive impairment, personality disorders, and epilepsy (Sellner and Trinka, 2012; Fruchter et al., 2015; Klein et al., 2017). It is vital to notice that the development of epilepsy has been reported 8 years after the onset of the encephalitis, and in nearly 60% of the patients infected with HSV (Sellner and Trinka, 2012; Bonello et al., 2015). *In vitro* studies have shown that HSV-1 is capable of upregulating the processing of amyloid-beta precursor protein (AβPP), increasing Aβ, suggesting the possibility that the infection with HSV can contribute to Alzheimer’s Disease (AD) (Shipley et al., 2005; Harris and Harris, 2018). Further, HSE in infants and adults promotes an alteration mediated by the immune response, where anti-NMDAR antibodies were detected after the HSV-1 infection (Leypoldt et al., 2013). This phenomenon was also associated with CHIKV infection (Nóbrega et al., 2020). Studies performed on mice infected with HSV-1 have shown a decrease in the NDMAR levels on hippocampal neurons, which induce symptoms such as depression, anhedonia, and memory deficits (Klein et al., 2017). The decrease of NMDAR is due to the increase of anti-NMDAR antibodies during HSE, and the consequences of these antibodies can be appreciated from the first 2 months and up to 9 months after the initial infection (Ewida et al., 2019). In some rare cases, as seen for ZIKV, HSVs can cause microcephaly on the offspring of infected mothers (Panchaud et al., 2016). Because HSV infection can cause a variety of sequelae, which can be cognitive-, psychiatric-, or neurodegenerative-related, patients infected with this virus need to be appropriately monitored for an extended period after the infection to manage the sequelae correctly (Figure 2; Fruchter et al., 2015; Klein et al., 2017).

Other viral infections associated with neurodegeneration are HIV, Influenza virus, and possibly SARS-CoV-2 (Chiara et al., 2012; Rogers et al., 2020). Infection with HIV induces HAND, causing cognitive impairment, dementia, and epilepsy (Kellinghaus et al., 2008; Kranick and Nath, 2012). Studies have indicated that 67% of the patients infected with HIV develop epilepsy, and in the case of infants infected with HIV, epilepsy has been associated with brain development problems and cognitive impairment (Kellinghaus et al., 2008). Several reports indicate that infection with HIV contributes to the onset of AD through the dysregulation of signaling pathways that would increase Aβ (Canet et al., 2018). Infection with neurotropic and non-neurotropic Influenza viruses may promote several neurologic sequelae, such as cognitive impairment, anxiety behavior, learning difficulties, and memory problems, as studies performed on mice have demonstrated (Hosseini et al., 2018). It was recently shown that the sequelae observed in mice infected with Influenza virus were caused by the alteration of function and morphology of the CA1 hippocampal neurons, increases in the glial cell density, and the activation in the hippocampus region (Hosseini et al., 2018). Furthermore, a later study suggested that non-neurotropic Influenza viruses can promote an increase in AD symptoms due to the stimulation of the systemic immune response that causes microglial hyperactivation (Figure 2; Hosseini et al., 2021).

Infection with hRSV in infants has been shown to induce language learning impairment, with children being unable to differentiate native phonetic, non-native phonetics and develop communication abilities (Peña et al., 2020). Moreover, studies on mice infected with hRSV showed that infection promotes learning impairment and behavioral alteration, which could be observed up to 60 days post-infection (Figure 2; Espinoza et al., 2013; Bohmwald et al., 2021b). Similar sequelae are reported for patients infected with coronaviruses, exhibiting symptoms such as impaired memory, depression, anxiety, and post-traumatic stress disorder (Rogers et al., 2020). Reports of patients infected with SARS-CoV-2 showed delirium and dysexecutive syndrome symptoms, a neurodegenerative disease affecting the frontal lobe of the brain (Poletti et al., 2017; Rogers et al., 2020). Further, the increase of pro-inflammatory cytokine response characteristic of the infection might be associated with the development of epilepsy in patients infected with SARS-CoV-2 (Nikbakht et al., 2020). Interestingly, a recent report showed that a patient diagnosed with encephalitis caused due to infection with SARS-CoV-2 had anti-NMDAR antibodies, which could suggest that the depression and memory-impaired symptoms are originated through a similar mechanism as the one described for HSV (Figure 2; Klein et al., 2017; Alvarez Bravo and Ramió i Torrentà, 2020).

## Conclusion

Encephalitis is a public health problem that has been increasing over the years either due to the emerging viruses, better diagnosis methods, or new knowledge about the etiology of the disease. Some of these emerging viruses causing encephalitis are considered to have an origin within animals and can spread the infection toward humans. Regarding viral encephalitis, it is clear now that not only the known neurotropic viruses such as WNV, TBEV, RABV, HSV, and HIV, are responsible for neuroinflammation, but also emerging respiratory viruses can cause this pathology (Granerod and Crowcroft, 2007; Chia and Hann Chu, 2013; Bohmwald et al., 2018; Yuan et al., 2019). During viral infections, microglial cells are the first line of defense and are the resident immune cells that initiate the local immune response. Once microglia are activated, astrocytes can also be activated and participate in the immune response along with microglia by the secretion of soluble factors such as cytokines and chemokines, inducing the recruitment of circulating immune cells into the injured zone of the brain (Colombo and Farina, 2016; Soung and Klein, 2018; Mangale et al., 2020). In this process, neurons can also amplify the immune response (Klein et al., 2017). The brain immune response can be altered when a virus infects either microglia, astrocytes, or neurons, increasing the inflammatory state and cell damage, which can translate from neurocognitive and motor alterations to the development of anti-NMDAR encephalitis or death (Huang et al., 2006; Morfopoulou et al., 2016; Susanne, 2018; Clé et al., 2020; Moriguchi et al., 2020; Pöyhönen et al., 2021). This phenomenon has also been seen in non-neurotropic viruses, which can induce systemic inflammation, capable of altering the BBB permeability, allowing the infiltration of immune cells that can directly impact brain cells or produce pro-inflammatory factors (Jurgens et al., 2012; Maria et al., 2018). Here, we discussed the current knowledge about the long-term sequelae of viral infections. Among the different consequences of a viral infection, neurodevelopmental delay, depression, cognitive disorders, memory impairment, language acquisition alteration, and ADHD can be found (Peña et al., 2020; Rogers et al., 2020; Sinanović, 2021). These neurological alterations can go unnoticed in most cases, which underscores the importance of increasing the number of studies evaluating the effects of viral infections with or without neuroinvasive characteristics.

## Author Contributions

All authors listed have made a substantial, direct, and intellectual contribution to the work, and approved it for publication.

## Conflict of Interest

The authors declare that the research was conducted in the absence of any commercial or financial relationships that could be construed as a potential conflict of interest.

## Publisher’s Note

All claims expressed in this article are solely those of the authors and do not necessarily represent those of their affiliated organizations, or those of the publisher, the editors and the reviewers. Any product that may be evaluated in this article, or claim that may be made by its manufacturer, is not guaranteed or endorsed by the publisher.
